# Highly multiplexed spatial profiling with CODEX: bioinformatic analysis and application in human disease

**DOI:** 10.1007/s00281-022-00974-0

**Published:** 2022-11-21

**Authors:** Wilson Kuswanto, Garry Nolan, Guolan Lu

**Affiliations:** 1 Department of Medicine, Division of Immunology and Rheumatology, Stanford University School of Medicine, Stanford, CA 94304 USA; 2 Department of Microbiology and Immunology, Stanford University School of Medicine, Stanford, CA 94304 USA; 3 Department of Pathology, Stanford University School of Medicine, Stanford, CA 94304 USA; 4 Department of Otolaryngology, Stanford University School of Medicine, Stanford, CA 94304 USA

**Keywords:** CODEX, Spatial profiling, Tissue immunology, Cell segmentation, Spatial analysis, Cellular neighborhoods

## Abstract

Multiplexed imaging, which enables spatial localization of proteins and RNA to cells within tissues, complements existing multi-omic technologies and has deepened our understanding of health and disease. CODEX, a multiplexed single-cell imaging technology, utilizes a microfluidics system that incorporates DNA barcoded antibodies to visualize 50 + cellular markers at the single-cell level. Here, we discuss the latest applications of CODEX to studies of cancer, autoimmunity, and infection as well as current bioinformatics approaches for analysis of multiplexed imaging data from preprocessing to cell segmentation and marker quantification to spatial analysis techniques. We conclude with a commentary on the challenges and future developments for multiplexed spatial profiling.

## Introduction

Microscopy-based technologies enable three-dimensional anatomic profiling at single-cell resolution, most often aided by visualization of cellular constituents. Determining the tissue architecture of multiple cell types, cell subsets, and their activation states requires the simultaneous detection of many cellular makers. Traditional immunofluorescence microscopy is usually limited to detection of four markers due to spectral overlap. Advances in multiplex tissue imaging have allowed for the simultaneous spatial detection of 50 + cellular proteins and up to 100 RNA markers with single-cell resolution [1]. Thus, multiplexed tissue imaging can now localize multiple immune, stromal, and epithelial cell types and subsets allowing mapping of tissue architecture and characterization of multicellular interactions. Though not covered in this review, additional advances allow as well the incorporation of genomic information to overlay on more traditional imaging techniques to provide deep interrogation of tissue functions.

**Fig. 1:**
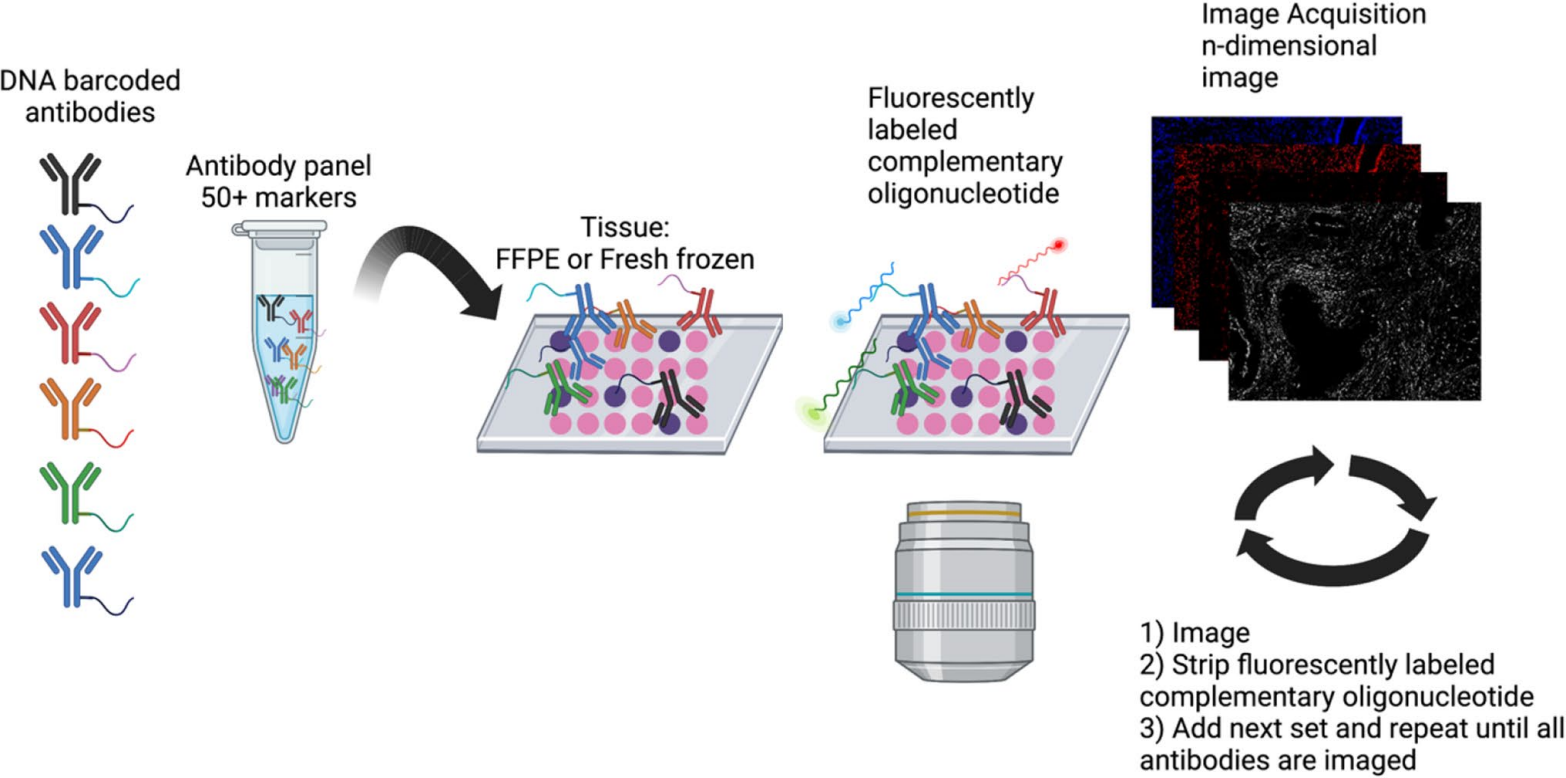
CODEX workflow. Fresh frozen tissue or formalin-fixed, paraffin-embedded (FFPE) tissue is stained with an antibody panel consisting of antibodies conjugated with unique DNA barcodes. Tissue is then stained with three complementary DNA oligonucleotides conjugated to fluorescent dyes. After imaging, the oligonucleotides are stripped off. The next set of fluorescent conjugated complementary DNA oligonucleotides are added, and the tissue is imaged. This cycle of oligonucleotide addition and stripping is iterated until all antibodies from the panel have been imaged

Several platforms for multiplexed tissue imaging have been developed over the past 5 years. Imaging Mass Cytometry and Multiplexed Ion Beam Imaging (MIBI) utilize antibodies conjugated to metal tags to spatially resolve single cells by mass cytometry and involves a single round of cellular staining and image acquisition [2, 3]. Other methods for multiplexed imaging incorporates cyclic fluorophore detection with repeated cycles of tissue staining, reporter/barcode stripping, image acquisition, and in the case of tissue-based cyclic immunofluorescence (t-CyCIF) [4] include multiple cycles of fluorophore bleaching. Co-Detection-by-InDEXing (CODEX) [5, 6] employs antibodies conjugated to barcoded oligonucleotides and is compatible with formalin-fixed, paraffin-embedded and with fresh frozen samples that are then stained with a panel of DNA barcoded antibodies [7]. Three fluorescent-dye conjugated oligonucleotides complementary to the antibody barcodes are imaged at a time; then, the fluorescent oligonucleotides are stripped off, and three additional fluorescently labeled oligonucleotides complementary to different barcodes are bound and imaged (Fig. 1). This process is iterated until all antibodies in the panel are imaged.

As important as the technical advances in multiplexed tissue imaging are the advances in the bioinformatic methods used to process and analyze the high-dimensional single-cell data to infer biological and clinical insights. Recent developments in computational analysis enable fast image preprocessing, robust cell segmentation, accurate protein marker quantification, followed by cell type identification and spatial analysis. These methods quantify marker expression in individual cells as well as the multi-scale spatial relationships to characterize tissue architecture and function.

In this review, we discuss use of CODEX to generate a healthy tissue cellular atlas and for spatial mapping of the immune, stromal, and epithelial landscape in cancer and autoimmunity (Table 1). Evaluating immune responses in tissues has revealed the wide variety of roles the immune system plays from organism-wide metabolism to tissue regeneration and repair and has highlighted the complex interplay among immune, stromal, vascular, nerve, and epithelial cells during physiologic and pathologic processes [8]. In the second part of this review, we outline the bioinformatic techniques (Table 2) that can be used to quantify and analyze the large datasets obtained from CODEX. It is notable that many of the techniques used for analysis of CODEX images can be applied to other imaging modalities and vice versa.

**Table 1:** Summary of current published studies utilizing CODEX

Manuscript	Disease state/tissue	Key Findings	Analytic tools
Ferreira et al., JCI Insight, 2021	Tissue injury/kidney	Distinct anatomic distribution of immune and epithelial cells after acute kidney injury	CODEX-MAV software
Phillips et al., Nature Comm, 2021	Cutaneous T cell lymphoma/skin	Distance between CD4 + PD1 + T cells, tumor cells, and Tregs, quantified by SpatialScore, correlates with response to checkpoint inhibitor	See SpatialScore in Table 2
Gouin et al., Nature Comm, 2021	Bladder cancer/bladder tumor	Identified CDH12 expressing epithelial tumor cells that predict response to immune checkpoint therapy	Cell types were identified by *k*-nearest neighbor and then manual gating. Niches were identified by *k* = 10 nearest cells
Schurch et al., Cell 2020	Colorectal cancer/colon	The two subsets of colorectal cancer has distinct cellular composition and organization. At the tumor boundary, the CD4^+^ T cell frequency and the CD4^+^ to CD8^+^ T cell ratio are prognostic indicators	See Cell Neighborhoods in Table 2
Mondello et al., Blood Cancer Journal, 2021	Follicular lymphoma, lymph node	Activated central memory T cells within tumor follicles are associated with improved prognosis	CODEX-MAV software for post-processing. Unet neural network for cell segmentation. Phenograph for cellular communities
Jiang et al., Front Immunol, 2021	Ebola infection/rhesus macaque spleen	Validated 21-marker panel to profile multiple immune cell types and Ebola virus in Rhesus Macaques	See CODEX toolkit in Table 2
Mayer et al., Research Square, 2021	Ulcerative colitis/colon	Identified inflammatory cell types and cellular neighborhoods that persisted despite treatment with TNFa inhibitors in ulcerative colitis. Developed a model utilizing spatial data to predict resistance to TNFa inhibitor therapy in ulcerative colitis	Utilized CODEX and cell neighborhood toolkits (see Table 2). For cell type identification, the authors performed unsupervised X-shift clustering with a manual gating strategy

**Table 2:** A summary of computational toolboxes for multiplexed imaging data analysis

Name	Preprocessing	Cell segmentation	Cell phenotyping	Spatial analysis	Language	Code source
RAPID	√				Matlab	https://github.com/nolanlab/RAPID
CODEX Toolkit	√	√			Java	https://github.com/nolanlab/CODEX
REDSEA	√				Matlab	https://github.com/nolanlab/REDSEA
CellProfiler		√			Python	https://github.com/CellProfiler/CellProfiler
Ilastik		√			Python	https://github.com/ilastik/ilastik
CellSeg		√			Python	https://michaellee1.github.io/CellSegSite/
Mesmer		√			Python	https://github.com/vanvalenlab/deepcell-tf
Cellpose		√			Python	https://github.com/mouseland/cellpose
CELESTA			√		R	https://github.com/plevritis-lab/CELESTA
STELLA			√		Python	https://github.com/snap-stanford/stellar
Astir			√		Python	https://github.com/camlab-bioml/astir
Cytofkit			√		R	https://github.com/JinmiaoChenLab/cytofkit
Cytomapper			√		R	https://github.com/BodenmillerGroup/cytomapper
ImaCyte			√	√	Matlab	https://github.com/biovault/ImaCytE
CytoMAP				√	Matlab	https://gitlab.com/gernerlab/cytomap/-/tree/master/CytoMAP
Cytokit	√	√			Python	https://github.com/hammerlab/cytokit
MCMICRO	√	√	√	√	Nextflow, Python	https://mcmicro.org/overview/
SIMPLI		√	√	√	Nextflow	https://github.com/ciccalab/SIMPLI
histoCAT				√	Matlab	https://github.com/BodenmillerGroup/histoCAT
SpatialScore				√	R	https://github.com/nolanlab/SpatialScore
Cell neighborhoods				√	Python	https://github.com/nolanlab/NeighborhoodCoordination
Spatial-LDA				√	Python	https://github.com/calico/spatial_lda
TissueSchematics				√	Python	https://github.com/nolanlab/TissueSchematics
MISTy				√	R	https://github.com/saezlab/mistyR

## Applications

### Molecular and spatial maps of the microenvironment of healthy and diseased tissues

A comprehensive roadmap of the molecular state of cells and its spatial localization in healthy human tissue provides the framework from which we can understand disease pathogenesis. Recently, multi-site consortia efforts have focused on the systematic study of diseased states, regulatory circuitry, and cellular interactions. CODEX has been utilized to generate detailed molecular and spatial maps of the cellular microenvironment of tissues from multiple organs in health and disease. The Human Cell Atlas Project is an international collaborative effort that aims to characterize the distinct molecular profiles of all cell types in the human body with spatial resolution [9]. The NIH Human Biomolecular Atlas Program (HubMAP) consortium coordinates multi-omic platforms spanning genomics, epigenetics, transcriptomics, and metabolomics with spatial protein and RNA expression across eight different organs [10]. Within these efforts, CODEX has been used to provide single-cell spatial data with protein expression at steady state in intestinal and colonic tissue. The Human Tumor Atlas Network (HTAN) is another multi-site collaborative effort that is utilizing molecular and spatial profiling to understand the progression of healthy tissue from a pre-cancerous state to localized cancer to metastatic disease [11]. This collaboration applies multiple-omic modalities to study DNA, RNA, and epigenetics with spatial localization at the single-cell and bulk tissue levels in multiple disease states. The goal of HTAN is to provide a framework for future therapies as part of the NIH Cancer Moonshot Initiative. As part of HTAN, CODEX has delineated the immune tumor microenvironment in lung cancer to unravel the cellular and molecular progression from pre-cancerous lesions to cancer.

One such organ that has been under intense study of this nature has been the kidney. Single-cell transcriptional studies have revealed that multiple cell types reside in human kidneys at steady-state and that there are coordinated immune, epithelial, and stromal changes following acute kidney injury [12]. Renal injury tends to be focal rather than diffuse, and understanding the spatial distribution of renal immune, stromal, and epithelial cell types following injury has provided insight into the localized changes that occur during acute kidney injury. CODEX has been used to study tissue architecture at the molecular level in two murine models of acute kidney injury, ischemia/reperfusion injury and cecal ligation puncture. Ferreira et al. utilized a 12-maker antibody panel to delineate B cells, CD4 and CD8 T naïve and memory cells, macrophages, endothelial cells, and other leukocytes by CODEX. The authors identified immune-epithelial interactions within distinct functional anatomical areas of the kidney in both models. In the ischemia/reperfusion injury model, neutrophils were primarily localized in the outer stripe region of the renal medulla. In the cecal ligation puncture (CLP) model, natural killer (NK) cells were primarily localized to the outer stripe region of the renal medulla but were also detected in the renal cortex. Additionally, in the CLP model, migrating macrophages infiltrated the outer renal cortex and co-localized with proximal tubule epithelial cells [12]. These spatial insights revealed the coordinated immune response to renal injury in distinct anatomic locations. While beyond the scope of this review, multiple other organ types including the lung [13], stomach [14], and colon [15] have been under similar focused study, with considerable success, underscoring the merits of the approach and the discoveries yet to be made.

### The immune tumor microenvironment and spatial predictors of response

Cancer treatment has advanced over the past decade with the transition from reliance on surgery, chemotherapy, and radiation that broadly target cancer toward the use of therapies that enhance the endogenous immune system [16]. Immune checkpoint inhibitors that target programmed cell death protein PD-1, its ligand PD-L1, or cytotoxic T lymphocyte-associated protein CTLA-4 “release the breaks” of the immune system to stimulate immune responses that target cancer cells [17]. Despite the effectiveness of checkpoint inhibitor therapies, it is difficult to predict which patients will respond [17]. Differentiating checkpoint inhibitor responders from non-responders is essential both to avoid treatment with ineffective therapies and to limit the risk of developing complications from checkpoint inhibitor use such as immune-related adverse events [18]. It has long been the assumption that form, positioning, and structure of cell contexts will predict function. Similarly, dysfunction though at times in cancer apparently random, also will be expected to follow some “order.” Based on this, multiplexed imaging platforms have been deployed for characterizing the immune tumor microenvironment through correlation of disease states with the mapping of locations of immune, stromal, epithelial, and tumor cellular components augments the phenotypic data obtained from DNA and RNA sequencing and epigenetics.

One such opportunity for study was cutaneous T cell lymphomas (CTCL), which are aggressive skin cancers that derive from CD4^+^ T cells. Patients with CTCL have poor prognosis, and treatment options are limited [19]. However, a subset of CTCL patients respond well to PD-1 blockade with robust and durable immune responses. Phillips et al. performed CODEX using a 54-marker antibody panel to detect T and B cells, macrophages, NK cells, granulocytes, dendritic cells, epithelial cells, endothelial, lymphatics, and multiple checkpoint markers to identify features that predict response to PD-1 blockade in CTCL patients [19, 20]. Over 70 tumor regions from 14 patients with advanced CTCL were evaluated, and given that biopsies were readily accessible from the same patient at the skin surface pre and post treatment, there was a unique opportunity to study cellular contexts in this disease.

In this study, there were not significant differences in the numbers or frequencies of immune or tumor cells between responders and non-responders; however, spatial analysis revealed that the distances between PD1^+^CD4^+^ T cells, tumor cells, and regulatory T cells, a population of dominant immune suppressors, predicted response to anti-PD-1 treatment. The spatial relationships among PD1^+^CD4^+^ T cells, tumor cells, and regulatory T cells were quantified by a very simple and intuitive “spatial score.” Patients with a low spatial score, which indicates that PD1^+^CD4^+^ T cells are in closer proximity to tumor cells than to suppressive regulatory T cells, were more likely to respond to anti-PD-1 treatment than were patients with a higher spatial score [19]. Thus, incorporating spatial analysis into clinical practice would allow identification of CTCL patients likely to respond to immune checkpoint blockade.

Another example is bladder cancer. For most patients with bladder cancer, neoadjuvant chemotherapy and immune checkpoint blockade have significantly improved outcomes. Those patients who have tumor epithelial cells that express the cadherin CDH12 have poor outcomes but respond well to immune checkpoint blockade [21]. To understand the underlying mechanism, Gouin et al. performed CODEX using a 35-marker antibody panel to profile tumors from twenty-five patients with bladder cancer and identified 360,000 epithelial cells, 140,000 immune cells, and 90,000 stromal cells [21]. The CDH12^+^ epithelial cells expressed PD-L1 and PD-L2 and co-localized with CD8^+^ T cells, which may explain why these tumors are responsive to checkpoint inhibitors. The CD8^+^ T cells displayed an exhausted phenotype as indicated by expression of PD-1 and LAG3. Using an unsupervised algorithm to quantify the cellular neighbors of CDH12^+^ epithelial cells revealed multiple “cellular niches.” Niches associated with CDH12^+^ epithelial cells tended to have considerable immune infiltration, whereas immune infiltration was not observed in KRT13^+^ epithelial cell niches. Additionally, CDH12^+^ epithelial cells in niches with immune infiltration exhibited higher PD-L1 expression than KRT13^+^ epithelial clusters. This study demonstrated that spatial information obtained using CODEX can reveal the cellular basis of improved response to immune checkpoint blockade.

Spatial analysis with CODEX has also been used to characterize colorectal cancer. Based on histology, colorectal carcinoma can be split into at least two groups: a Crohn’s-like reaction (CLR) subset in which tertiary lymphoid structures are observed at the tumor invasive front and a diffuse inflammatory infiltration (DII) subset [7]. Patients with the CLR subset have significantly better prognosis than those with the DII subset. CODEX with a 56-marker panel was used to quantify immune, stromal, and epithelial cells in colorectal carcinoma tumors and unsupervised clustering identified two clusters corresponding to CLR and DII subsets based on the presence of tertiary lymphoid structures [7]. Decomposition of differences in cell types and cellular neighborhoods utilizing tensor decomposition revealed that there are distinct organizational differences in the immune tumor microenvironments of the two subsets. CD4^+^ T cell frequency and the CD4^+^ to CD8^+^ T cell ratio at the tumor boundary served as a prognostic indicator and suggested T cell activity at the tumor boundary is critical to a productive immune response [7].

In another example of use of CODEX to determine how spatial neighborhoods influence response to therapy, Mondello et al. evaluated samples from patients with follicular lymphoma, which is typically an indolent disease with a subset of patients who have early relapse with poor outcomes [22]. The immune tumor microenvironments of 496 diagnostic biopsies from patients with early follicular lymphoma who did not respond to treatment were evaluated. The follicular lymphoma immune tumor microenvironment was enriched for memory and naïve CD4^+^ and CD8^+^ T cells, B cells, monocytes/macrophages, and endothelial cells. The presence of activated central memory T cells within tumor follicles was associated with favorable outcomes and improved prognosis that was independent from genetic features [22]. Thus, combining spatial anatomy of follicular central memory T cells with clinical markers improved identification of high-risk patients.

### Spatial microenvironment in infection and autoimmunity

To understand host immune responses to pathogens in human and primate models requires extensive reagent development, and for pathogens that are deadly or highly infectious, optimization for use in inactivated samples may be necessary. A 21-marker CODEX antibody panel that delineates multiple immune cell types spanning granulocytes, plasma cells, and CD4^+^ and CD8^+^ T cells and B cells with three Ebola virus specific antibodies has been validated for study of host responses to Ebola virus [23]. This antibody panel has been validated in fully inactivated FFPE samples and highlights a platform that can be used to probe cellular immune responses to Ebola virus and other pathogens.

CODEX has also been used to study tissue from patients with ulcerative colitis, an autoimmune disease with relapsing and remitting inflammation in the large intestine [24]. Ulcerative colitis is heterogeneous with a subset of patients responding well to antibodies that block activity of TNFa [15]. CODEX has been used to characterize the inflammatory microenvironment and to identify features that predict response to TNFa inhibitors and overall prognosis. Mayer et al. profiled 29 patients with ulcerative colitis and five healthy controls with a 52-marker antibody panel to detect immune, epithelial, and stromal populations and identified 13 distinct cell types. In healthy controls, there was enriched contact between epithelial and stromal cells. In ulcerative colitis patients treated with TNFa inhibitors, there were decreased T cell interactions with other adaptive immune cells, stroma, and epithelium; however, there was no difference between T cell interactions with the innate compartment. Cell type neighborhood analysis revealed ten cellular neighborhoods that spanned primarily immune, mixed immune epithelial, primary epithelial, inflamed vasculature, stroma, and inflamed stroma. In responders, treatment with TNFa inhibitors resulted in a decrease in the adaptive immune tissue neighborhoods with a corresponding increase in healthy epithelial tissue neighborhoods while frequencies of innate immune predominant tissue neighborhoods did not change. A model utilizing CODEX spatial analysis incorporating cell types, cell–cell contact, and cellular neighbors was able to predict resistance to TNFa inhibitors and outperformed models that relied on transcriptional data alone [16]. Incorporating tissue spatial analysis with other clinical indicators could direct future therapeutic treatment algorithms.

## Bioinformatic analysis

**Fig. 2:**
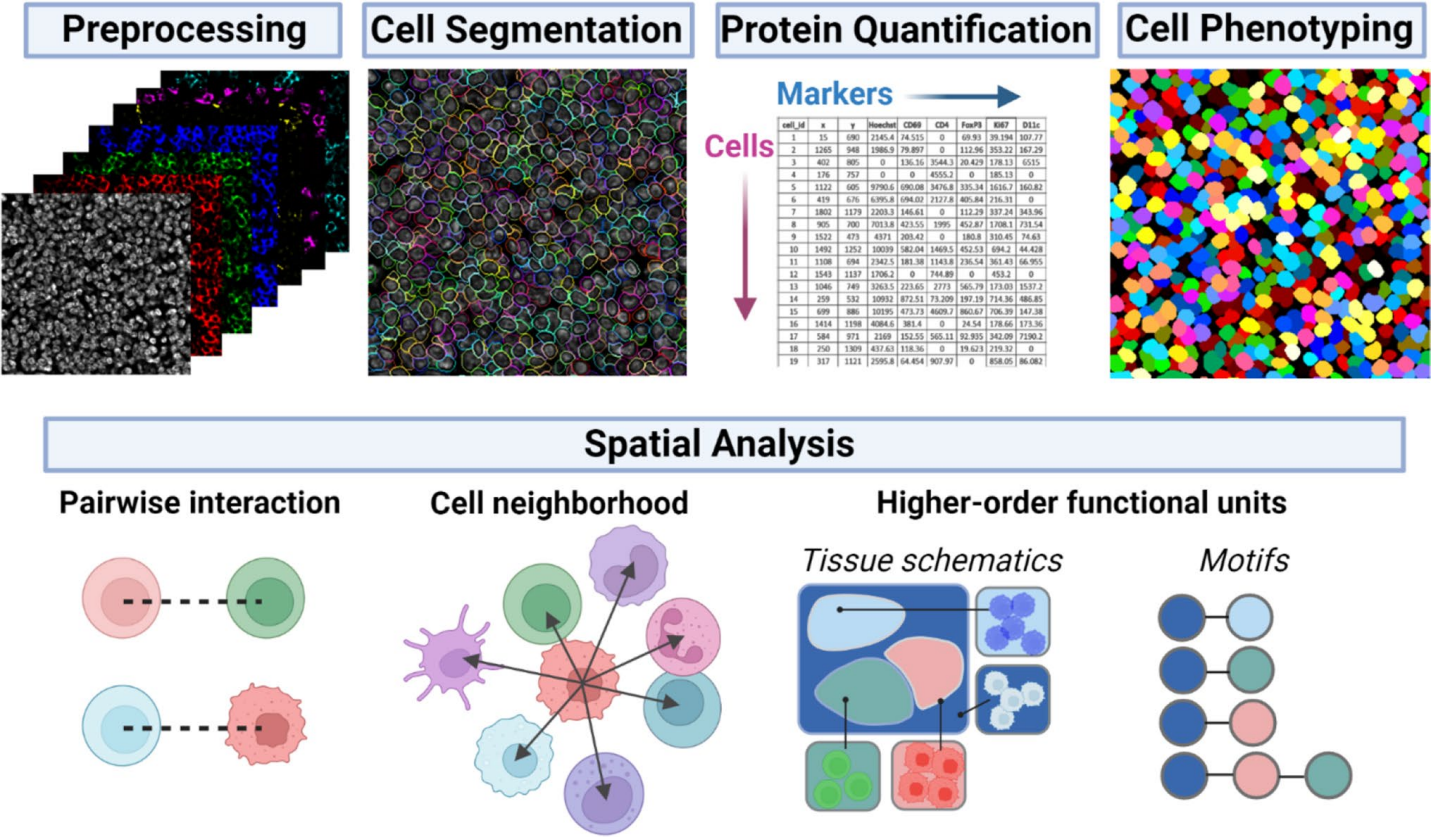
Overview of the computational workflow for CODEX multiplexed imaging data

Bioinformatics analysis of CODEX multiplexed imaging data reveals tissue structure and provides insight into biological mechanisms. This is typically done by converting the high-dimensional raw data into single-cell maps of tissue architecture and functional states. This process consists of four main steps (Fig. 2): (1) image preprocessing, (2) cell segmentation, (3) cell phenotyping, and (4) spatial analysis by one or more algorithms that relate cellular contexts to some determinative or predictive outcome.

Over the past 5 years, various computational methods and software tools have been developed to facilitate each of these analytical steps. Most carry out only one of the steps (CODEX Uploader [5], RAPID [25], CellSeg [26], Mesmer [27], CellPose [28], CELESTA [29], Astir [30], CytoMAP [31], histoCAT [32], TissueSchematics [33], and MISTy [34]), but a few attempt to address multiple steps or the entire workflow (Cytokit [35], MCMICRO [36], SIMPLI [37]) (Table 2).

### Image preprocessing

Typical CODEX multiplexed imaging data consist of multiple tiles that correspond to a large tissue region. In each cycle, data are collected from four wavelength channels and multiple z-planes, resulting in terabytes of imaging data for each experiment. To visualize and analyze this dataset, it is necessary to convert the raw data into multidimensional hyperstacks (*x*, *y*, channel, cycle) by creating montages from individual tiles and aligning these montages from different cycles to generate a multidimensional tiff image. Several challenges are associated with this step: First, images can be blurry and noisy due to inherent limitations of microscopes systems (e.g., out-of-focus light and noise from the light source and the camera). Second, images can be misaligned due to axial and lateral drift during the cyclic imaging process. Third, there is autofluorescence background originating from endogenous tissue components such as erythrocytes, collagen, elastin, and lipofuscin, and formalin fixation. Finally, rapid processing of the large-scale imaging datasets. These issues are generic to all fluorescence-based multiplexed imaging technologies including CODEX.

Although several computational toolboxes are available for CODEX image preprocessing, most only address a subset of these issues. The CODEX Uploader, the first processor developed for CODEX data, was implemented as an open-source Java package by the Nolan lab [5–7]. This software corrects spatial drifts using 3D drift-compensation, deconvolves the *z*-stack images using the commercially available Microvolution algorithm, subtracts the background obtained by collection of blank image cycles, and generates hyperstacks consisting of all fluorescent channels and imaging cycles. The CODEX Uploader has a graphical user interface (GUI) that allows users without any programming background to process the complex multiplexed imaging data. The main limitations of the CODEX Uploader are that it can take several days to process terabytes of imaging data and it does not correct lateral drifts among tiles for multi-tile experiments. Cytokit, which was implemented in Python, employs a pipeline similar to that of the CODEX Uploader [35]. MCMICRO can be used to analyze multiplexed imaging data collected with a range of technologies (e.g., fluorophore-based, metal-based) [36]. The preprocessing pipeline in MCMICRO includes illumination correction, image stitching, and registration, but it does not have image deconvolution or autofluorescence removal.

RAPID [25] was developed for accurate and fast analysis of large-scale CODEX imaging data by the Nolan lab. It reduces processing time by two to threefold compared to the CODEX Uploader. RAPID deconvolves large-scale CODEX imaging data and stitches and registers images with axial and lateral drift correction. Moreover, RAPID minimizes intense tissue autofluorescence such as that introduced by erythrocytes, thereby improving the immunofluorescence detection of antigens, especially for low-abundance markers. RAPID incorporates an open-source, CUDA-driven, GPU-assisted deconvolution algorithm rather than the fee-based commercial software used in the CODEX Uploader with no impact on quality of the output.

### Cell segmentation

After the raw imaging data is processed and aligned, the next step is cell segmentation. During this step, the boundaries of single cells are computationally identified and binary masks for individual cells are generated. This task has long been challenging in fluorescence microscopy image processing. While it is relatively easy to segment monodispersed cells in images of cultured cells as cells are sufficiently separated, segmenting cells that are densely packed in tissues such as lymph nodes and spleen is difficult. The accuracy of cell segmentation significantly influences the quantification of multicellular properties such as protein expression and cell morphology. An ideal cell segmentation algorithm for imaging data must accurately segment cells of various sizes and shapes independently of density across diverse tissue types and must be capable of demarcating the membrane, nucleus, and cytoplasm.

Cell segmentation algorithms typically use images of cells stained with marker of nuclei and/or membranes as input. Some algorithms generate only nuclei masks, but others can output both nuclei and whole-cell masks, thereby allowing protein quantification in sub-cellular compartments. Watershed nuclei segmentation algorithms are classical segmentation methods that perform well when segmenting cells of relatively homogenous size and shape such as lymphoid tissue in CODEX data [5, 6, 38]. However, watershed-based algorithms can be sensitive to noise such as imaging artifacts and blurred cell boundaries. In addition, significant tuning for multiple user-defined parameters based on the cell morphology and image intensity in the tissue images is necessary. Therefore, watershed cell segmentation does not work well for tissues that have cells of diverse sizes, shapes, and densities such as those in the tumor microenvironment.

Machine learning and deep learning algorithms trained on large-scale datasets are more robust to variations of image quality and generalize better across tissue types than watershed-based algorithms. Machine learning and deep learning algorithms differ in the composition and quantity of training dataset, model architecture, outputs, and post-processing methods. CellSeg is a modified Mask R-CNN algorithm that accurately segments nuclei in CODEX images and outperforms watershed-based nuclei segmentation [26]. CellSeg showed robust performance in delineating nuclei boundaries even in noisy images with low signal-to-noise ratio. A limitation is that it approximates the whole-cell mask by expanding the nuclei mask for a few pixels, which may not accurately match the cell boundaries. Cellpose is designed as a generalist algorithm for cell segmentation that is capable of generating both nuclei and whole-cell masks [28]. Although Cellpose outperformed several deep learning-based cell segmentation algorithms including Mask R-CNN, Stardist, and standard U-Nets, none of these models were trained on a multiplexed tissue imaging dataset; instead, training was implemented on cultured cells, animal cells, and non-microscopy images of non-cell objects. Recently, Noah et al. constructed a large-scale multiplexed imaging dataset named TissueNet with more than 1 million manually labeled cells [27]. Using TissueNet, they trained a deep neural network named Mesmer and achieved human-level performance in segmenting whole cells. Mesmer outperformed the pre-trained Cellpose model, FeatureNet, RetinaMask, and ilastik [39], and enabled both nuclei and whole-cell segmentation, thereby allowing for subcellular localization of protein signals. Interestingly, different deep learning models achieved similar performance when trained on the same TissueNet dataset, suggesting the importance of procuring large-scale annotated dataset.

### Protein marker quantification

After cell segmentation, protein marker expression is typically quantified as the total marker intensity on each cell normalized by cell size. However, spatial spillover, which is the blending of signals between neighboring cells that are tightly packed such as in lymphoid tissue, is a common issue complicating the accurate quantification of single-cell protein expression in multiplexed imaging data. Signal spillover is often manifested by the false positive co-expression of mutually exclusive markers on the same cell. An example is the apparent co-expression of CD3 and CD20, which are expressed on T and B cells, respectively. To compensate for the cell-to-cell spillover in CODEX images, an adjacency matrix is typically calculated by measuring the fraction of shared boundary between each pair of cells, and then the raw intensity matrix of protein expression is multiplied by the inverse adjacency matrix, correcting for the spillover [5]. REDSEA is another spillover correction method that has been shown to decrease signal contamination from neighboring cells in MIBI data [40]. However, REDSEA requires that the cell mask have zero-pixel value between the adjacent cell boundaries, which is not the case with the typical cell masks generated by commonly used cell-segmentation algorithms.

### Cell type identification

Assignment of cell types, namely, cell phenotyping, to segmented single cells from multiplexed imaging data remains a bottleneck for multiplexed image analysis. An ideal algorithm should automatically and precisely define cell types and subtypes of various abundances in an objective and robust manner with minimal human intervention.

The classical methods for cell phenotyping rely heavily on pathology expertise and are subjective and time-consuming. One such method employs the manual gating strategy that is typically used for analyzing flow cytometry or CyTOF datasets (e.g. Cytofkit [41], cytomapper [42]). This method is sensitive to signal spillover between adjacent cells and to segmentation noise [43]. SIMPLI [37] is a toolkit that defines cell types by global thresholding in a fashion similar to the hand-gating method; both are challenged by poor imaging quality and imaging noise.

Unsupervised clustering followed by manual annotation has been widely used for assignment of cell types [5, 7, 19]. This process typically starts with over-clustering of a protein expression matrix with each cell as a row and each marker signal as a column. Many clustering algorithms are available that can be used to group protein expression into clusters; these include X-shift, PhenoGraph, FlowSoM, and *k*-means, etc. Data normalization techniques and selection of the optimal cluster numbers can significantly influence the clustering results [43]. After the initial clustering, similar clusters are merged, and mixed clusters are further separated to finalize the cluster annotation. This process works in a bottom-up and iterative fashion. However, problems such as mixed clusters or unknown clusters can persist after many rounds of manual annotations, and annotating millions of cells requires significant time [7].

Although efforts have been made to develop new algorithms for automatic and fast cell type identification, robust and accurate algorithms that can detect cells of various abundances from multiplexed imaging data are still lacking. Astir [30] is a probabilistic machine learning method that infers cell types based on protein expression of cells and prior knowledge of marker proteins. This algorithm has superior accuracy and runtime compared to unsupervised clustering methods in mass cytometry data. However, it did not outperform supervised classification and cannot identify novel cell types. Additionally, Astir is sensitive to antibody staining quality and signal intensity. Although Astir utilizes protein expression profiles, it does not incorporate any spatial or morphological information unique to multiplexed imaging data. CELESTA is a fast cell-identification algorithm designed to leverage both the protein expression and spatial information from CODEX imaging data [29]. Cells with marker expressions that matches well with the pre-defined marker profile are easily identified and defined as “anchor” cells. When the marker expressions of cells do not match a pre-defined cell type, these cells are assigned an identity based on a spatial score of their neighboring cells types. One limitation of this method is that it is difficult to define rare cell types. CELESTA is also sensitive to marker staining quality and imaging noise. STELLAR [44] is new cell-type annotation method that uses graph convolutional neural network to learn the spatial and molecular similarities of cells. This method requires manually annotated data as a reference dataset to learn from.

### Spatial analysis

Multiplexed imaging data contains rich information on the multicellular tissue ecosystem including cell types, cell states, and their coordinated activities across scales. The goal of spatial analysis is to decipher how cells and tissues are spatially organized and orchestrated in their native environment and how this organization influences biological function, disease progression, and response to therapies. To detangle this multilayered and interrelated spatial information, computational methods must dissect the multicellular modules that are coordinated by both the physically proximal and distant cell types across a spectrum of length scales and determine the associations of these multicellular modules with tissue functions and with physiological and pathological conditions.

Cell–cell interactions and communications can occur across a range of distances [45], broadly categorized as autocrine, juxtacrine, paracrine, and endocrine. The developed spatial analysis approaches span from identification of pair-wise cell–cell contacts to higher-order architectures.

Pair-wise cell–cell interactions: The most basic method for cell interaction analysis is to measure the pair-wise cell–cell interactions to infer attraction or repulsion between two cell populations. To measure these physical cell–cell contacts (i.e., juxtacrine contacts), Goltsev et al. identified pairs of neighboring cells from Delaunay graphs and calculated the odds ratio of the co-occurrence of two cell types [5]. This analysis showed that the most dominate pairwise cell–cell contacts are homotypic (e.g., T cells with T cells, B cells with B cells) and mostly reflect anatomic compartments in the tissue [5, 7]. histoCAT was originally designed for cell-interaction analysis in multiplexed image cytometry data [32]. It examines the significance of pairwise interactions by comparing the number of cell–cell interactions at a user-defined distance (e.g., 4 pixels) to that of a matched control containing randomly shuffled cell phenotypes [46]. This test can reveal significant enrichments or depletions of a cell in another cell’s neighborhood. Strategy used by Keren et al. [3] measured pair-wise interactions within 100 pixels (39 µm) of an index cell. Similar to the method developed by Goltsev et al., these pair-wise enrichment analysis [3, 46] identified mostly homotypic interactions between similar cells, suggesting that this might be a limitation of pair-wise analysis. Additionally, several spatial descriptive functions, including the K-function, L-function, and pair correlation function, can also be used to evaluate the spatial relationships between two cell populations [47].

Cell neighborhood, niche, or community analysis: Cell neighborhood analysis identifies higher-order (rather than paired) interactions between one or more cell phenotypes, which provides a catalog of repeating architectural units for tissues. Many methods are available to identify cell neighborhoods. A neighborhood is defined as a locally spatially homogeneous mixture of cell types [33]. The neighborhood identification methods typically raster scan each cell across the tissue with a pre-defined window that consists of *N* nearest neighboring cells (e.g., *N* = 10 was found to be suitable for colorectal tumor microenvironment [7]) or all the cells within a certain physical distance of the center cell, which is the strategy used in CytoMAP [31]. Jackson et al. used a graph-based method to identify spatially connected cell clusters, which they called cell communities [46]. These windows or graphs are grouped into cell neighborhoods by unsupervised clustering of the cell type composition of the windows. The size of these windows influences the type of cell neighborhoods identified. Spatial-LDA [48] is another method to identify cell neighborhoods with smooth boundary transitions, but it is very slow for large image data. Cell neighborhoods are composed of distinct spatial patterns of cell organization, thus reflecting characteristic local processes. Some neighborhoods correspond to known tissue microanatomies, such as tertiary lymphoid structure and bulk tumors, whereas others are novel such as the granulocyte-enriched cell neighborhood identified by Schürch et al. in their analysis of colorectal tumors [7]. In addition, other computational methods have been used to identify patterns of cell types and cell neighborhoods from multiplexed imaging data. For example, non-negative Tucker tensor decomposition and canonical correlation analysis have been used to evaluate cell type and cell neighborhood interactions [7]. “SpatialScore” was shown to predict immunotherapy response by measuring the physical distance ratio of each CD4 + T cell and its nearest tumor cell relative to its nearest Treg [19]. The tumor-immune mixing score is another spatial metric that has been used to quantify the degree of mixing between tumor and immune cells [3].

Higher-order functional units: It remains understudied how cell neighborhoods are assembled into higher-order, repetitive structures that coordinate complex tissue functions and promote disease development and progression. To infer the design principles of spatial organization of tissues, Bhate et al. proposed a conceptual and analytical framework to define and quantify tissue architectural units and their assembly at both the local and global level from multiplexed imaging data like CODEX [33]. Development of the framework, which Bhate et al. called the tissue schematic, starts by identification of cell neighborhoods from recurring patterns of spatially proximal cell types, next a graph is constructed using cell neighborhoods spanning the entire tissue sections, and finally recurring cell neighborhood combinations are classified as “motifs.” The junctions between two cell neighborhood boarders are the sites of possible interactions between the two local tissue processes; thus, “motifs” could have emergent functionality arising from signal propagation between cellular neighborhoods. Applied to CODEX imaging data of lymphoid tissues, tissue schematics identified the assembly rules shared by tonsils, lymph nodes, and spleen. In addition, this method revealed that the insertion of a tumor cell neighborhood into assembled regions of the T cell-enriched, macrophage-enriched, and vasculature neighborhoods was associated with poor survival outcomes. These results suggested the basic and translational utility of tissue schematics to understand tissue function and malfunction.

Spatial patterns of protein distribution: In addition to the geospatial distribution of cells in tissues, the expression levels and spatial patterns of protein markers are also important indicators of tissue functions. Goltsev et al. showed that cell surface marker expression is dependent on immediate neighbor cells [5]. For example, levels of CD79b and B220 are niche dependent in spleens of mice. These data suggested that geospatial location of cells within a tissue could be an indicator of potential functions. Keren et al. found that the expression of immunoregulatory proteins in distinct cell types correlates with the tissue architecture (mixed and compartmentalized) and their distance to tumor-immune borders in MIBI of human breast cancer tissue [3]. Recently, MISTy, a machine learning framework to quantify and predict the relationships between marker expression and the local cellular neighborhood (juxtaview) and broader tissue structure (paraview) was introduced [34]. Interestingly, using this algorithm to analyze imaging mass cytometry data of human breast cancer tissue, Tanevski et al. showed that broader tissue structure has more effect on the protein marker expression than do immediate neighbors.

## Challenges and future directions

The past half-decade has witnessed rapid technological development and increasing biomedical applications of highly multiplexed imaging technologies including CODEX. These technologies quantify cell types and states and reveal the spatial organization and coordinated actions of cell types and multicellular modules in the native microenvironments of healthy and diseased tissue. In this short review, we discussed how application of CODEX has improved our understanding of the immune tumor microenvironment and has identified features that are predictive of prognosis and response to immune checkpoint inhibitors. For diseases that encompass autoimmunity, CODEX has been used to spatially map the immune, stromal, and epithelial cell types involved in ulcerative colitis and has identified a cellular neighborhood that did not resolve after treatment with TNFa inhibitors. Though the number of studies on autoimmunity and host–pathogen response are limited relative to cancer studies, we expect that in the upcoming years, multiplexed technologies will delineate the complex interactions between multiple cell types in various autoimmune diseases such as type 1 diabetes and in host–pathogen responses to viruses such as SARS-CoV-2. An inherent difficulty with fluorescence based multiparameter imaging is autofluorescence. Despite techniques to limit autofluorescence such as photobleaching or post-processing tools, certain tissues, for example bone marrow or cartilage, remain difficult and mass spectrometry-based platforms may be more suitable.

The newest CODEX multiplexed imaging system enables imaging of 100 markers, compared to 50 with the earlier system, and the imaging time has also been reduced. With these technological advancements, high-plex, large-scale multi-omic datasets for various diseases will be generated. However, computational methods that can effectively convert these datasets into single-cell maps of tissue architecture and function are still lacking. Although CODEX image processing and cell segmentation methods are available, computational methods are needed to address several major issues. First, automatic cell type identification methods that are accurate, fast, and robust to imaging noise and staining intensity to result in high-quality and have multi-level granularity for cell phenotypes must be developed. Second, spatial analysis methods must be created that can identify the multicellular modules coordinated by both the physically proximal and distant cell types across a spectrum of distances in the same sample or patient.

Although not reviewed here, new technologies for directly visualizing RNA at the single-cell resolution are being developed by several academic and commercial groups. Recently a method called PANNINI [49] has been developed and integrated with MIBI to visualize viral DNA, RNA, as well as protein makers. This opens up new opportunities for simultaneous multiplexed nucleic acid and protein imaging in situ. In addition to technological advancement, new computational methods are being developed to integrate and match complementary multimodal information (e.g. proteomics, transcriptomics, epigenetics, genomics, and metabolomics) cross tissue (e.g. spleen to tonsil) and cross species (e.g. non-human primates to humans), thereby providing a systems-level understanding of biological processes. One such method named MARIO [50] is capable of integrating single-cell RNA sequencing data (CITE-seq) with multiplexed single-cell protein imaging (e.g., CODEX, MIBI), which could link protein states to gene regulatory states.

Multiplexed imaging studies have uncovered cell–cell interactions associated with disease conditions. We envision that future effort will focus on:

1. Measuring these multicellular interactions and functions with high temporal resolution in three-dimensional space. Experimental and computational approaches need to be developed to dissect the multicellular communications at higher spatial, temporal, and molecular resolution.
2. Designing basic mechanistic studies to identify causal relationships beyond the correlative studies. We predict that in the next few years, researchers will perturb the cellular microarchitecture to determine the functions of particular cell subsets and cellular neighborhoods or motifs. Efforts from these studies will enable the development of more accurate in silico and in vitro models such as organoids or systems-on-a-chip.
3. Translating predictive and prognostic biomarkers to improve patient outcomes in the clinic. As the number of studies from CODEX and other multiparameter imaging platforms increase in throughput, we predict that we will better understand human disease heterogeneity. Prospective clinical studies will be conducted to validate the clinical utility of these biomarkers in large-scale patient populations.

In the future, multiparameter imaging could be incorporated to stratify patients with diseases such as rheumatoid arthritis, ulcerative colitis, and cancers. Although biologics have greatly improved patient health in the treatment of various autoimmune diseases, identifying which biologic class will be effective in which patient has relied on a “trial and error” approach. We anticipate that as high-throughput, multiparameter imaging modalities are incorporated into hospital laboratories, disease features derived from multiplexed imaging that are predictive of treatment response could be incorporated into clinical guidelines.
